# Unraveling the Specific Recognition Between PD-L1 and Engineered CLP002 Functionalized Gold Nanostructures: MD Simulation Studies

**DOI:** 10.3390/molecules30092045

**Published:** 2025-05-04

**Authors:** Micaela Giannetti, Marina Gobbo, Lucio Litti, Isabella Caligiuri, Flavio Rizzolio, Moreno Meneghetti, Claudia Mazzuca, Antonio Palleschi

**Affiliations:** 1 Department of Chemical Science and Technologies, University of Rome “Tor Vergata”, Via della Ricerca Scientifica, 00133 Rome, Italy; 2 Department of Chemical Sciences, University of Padova, Via F. Marzolo 1, 35131 Padova, Italy; marina.gobbo@unipd.it (M.G.); lucio.litti@unipd.it (L.L.); moreno.meneghetti@unipd.it (M.M.); 3 Pathology Unit, Centro di Riferimento Oncologico di Aviano (CRO) IRCCS, Via F. Gallini 2, 33081 Aviano, Italy; 4 Pathology Unit, Department of Molecular Sciences and Nanosystems, Ca’ Foscari University of Venice, Via Torino 155, 30172 Venice, Italy

**Keywords:** peptide monolayer, immune checkpoint, molecular dynamic simulations, SERS, gold nanoparticle

## Abstract

PD-L1 (programmed cell death ligand-1) is a protein located on the surface of regulatory cells. It has an immunosuppressive role as it binds specifically to the protein programmed cell death-1 (PD-1), a checkpoint glycoprotein, present on the surface of immune cells such as T and B lymphocytes. Many tumor cells block the immune response by overexpressing PD-L1 on their surface; therefore, targeting PD-L1 represents a powerful strategy that allows tumor localization. To determine the presence of PD-L1 in cells, the use of ad hoc functionalized peptides that bind to PD-L1 can be exploited. One of them is the peptide CLP002 (Trp-His-Arg-Ser-Tyr-Tyr-Thr-Trp-Asn-Leu-Asn-Thr), which, bound to surface-enhanced Raman scattering (SERS) gold nanostructures via a suitable linker, was shown to be highly effective in recognizing MDA-MB-231 breast cancer cells and, importantly, this recognition can be measured by SERS experiments. To characterize, on a molecular scale, the interaction between PD-L1 and peptide functionalized nanostructures, we performed molecular dynamics (MDs) simulations, studying the features of peptide monolayers bound on gold surfaces in the absence and presence of PD-L1. The results obtained allow us to explain why the nature of the linker plays a fundamental role in the binding and why a peptide carrying the same amino acids as CPL002 but with a different sequence (scrambled) is much less active than CLP002. These results open the way to an in silico evaluation of the key parameters that regulate the binding of PD-L1 useful for cancer recognition.

## 1. Introduction

Chemotherapy faces significant challenges because of its inability to selectively target cancer cells. Currently, these treatments attack all rapidly dividing cells, affecting both malignant and healthy tissues. To limit severe side effects in patients, the treatment dose is often restricted, with there being a risk of leaving cancer cells alive and promoting drug resistance [1,2,3]. Therefore, there is a pressing need for cancer therapies that are more fine-tuned and less harmful, thus achieving greater therapeutic and diagnostic precision with minimal adverse effects [4,5,6]. A promising strategy lies in the development of nanotechnology with theragnostic capabilities. Theragnostic nanomedicine integrates functions for diagnosis, targeted therapy, and monitoring the response to the treatment [7,8,9]. In this context, in order to identify specific tumor cells with increased sensitivity and specificity, nanostructures (NSs) composed of gold nanoparticle (NP) aggregates, functionalized with ad hoc peptides designed to target specific receptors expressed on cancer cell surfaces, have recently been proposed [10,11,12,13]. NSs, in fact, display plasmonic properties, such as surface-enhanced Raman scattering (SERS), which are useful for the verification of tumor cell targeting [10,11,12,13]. Peptides have numerous advantages over small molecules and antibodies, yet they are used to treat a wide range of cancers [14,15,16]. In fact, peptides are characterized by low toxicity, reduced immunogenicity, high synthesis fidelity, and low production cost with well-standardized and reproducible procedures [17,18]. Moreover, the linking of peptides to nanoparticles to obtain an engineered coating allows us to overcome the following two drawbacks concerning the use of peptides for theragnostic purposes: (a) peptides have lower receptor affinity than antibodies; (b) they can be easily digested by proteolytic enzymes. This is because since the number of peptides in a single NP is in the order of several thousands the avidity of such NSs rivals that of antibodies and the enzymatic degradation of peptides is almost suppressed [11,12,19]. In recent research, we have shown that NSs functionalized with GE11 peptides are capable of efficiently targeting epidermal growth factor receptors, overexpressed by epithelial tumors, and that NSs functionalized with cyclic RGD peptides can be usable for imaging and targeting colorectal cancer cells [10,11,12]. The success of this targeting strategy is significantly dependent on the intricate engineering of these peptides in the functionalization of NSs. In fact, the presence and characteristics (that is, charge and length) of the linker between the gold surface and the peptide, as well as the copresence of PEG chains linked to NSs, play a crucial role in their targeting activity [12].

More recently, novel cancer treatment strategies have involved the targeting of PD-L1, a key immune checkpoint protein implicated in the ability of tumor cells to suppress immune responses [20,21,22]. More specifically, PD-L1 is a protein present in tumor cells which suppresses the immune response of T-cells by binding to the PD-1 receptor, present in T-cells (Kd ~ 7 μM) [23,24,25]. Tumor cells exploit this interaction as a strategy to escape the immune system. The presence of a high amount of PD-L1 in tumor cells represents a key factor in the establishment of both a smart targeting tool for cancer diagnosis and a strategy for cancer therapy; thus, targeting PD-L1 has become one of the most studied areas in cancer immunotherapy.

Currently, several PD-L1 inhibitors have been approved such as Nivolumab (Opdivo) and Avelumab (Bavencio) [26,27]. They work by blocking the interaction between PD-L1 in cancer cells and PD-1 in T cells. All these drugs are monoclonal antibodies that can lead to infusion reactions and immunogenicity and the production of antidrug antibodies (ADAs), potentially diminishing their therapeutic efficacy or causing adverse reactions. Furthermore, the cost of these therapies can be prohibitive, potentially limiting accessibility for some patients [28]. For these reasons, new modalities that offer improved therapeutic outcomes compared to existing therapies, including the use of peptides and nanostructures, have attracted significant interest due to their reduced immunogenicity, cost-effectiveness, and improved potential to penetrate tumors [29,30,31]. In this context, a good alternative is represented by the peptide CLP002 (Table 1), identified by bacterial surface display techniques, which binds to the PD-L1 antigen with a Kd ~ 0.4 µM [32,33].

Recently, we have shown that the programmed death ligand-1 (PD-L1) protein can also be successfully targeted by functionalized NPs with CLP002 carrying a suitable linker (Table 1), as demonstrated by SERS measurements after incubation with tumor cells [13]. Importantly, the specificity of functionalized NSs was verified by preparing NSs using a modified peptide with a scrambled sequence of CLP002, that is, SCLP002 (Table 1). These NSs are much less active toward PD-L1. Furthermore, we have experimentally found that the primary sequence is not the only crucial factor in the targeting activity of functionalized and that the type and length of peptide linkers to NPs are also crucial [13]. In this article, molecular dynamics (MDs) studies were performed to elucidate the key molecular characteristics that govern effective NS-peptide/PD-L1 binding, based on an analysis of peptide monolayers attached to gold surfaces in both the presence and absence of PD-L1. It is a true step forward in the characterization of these systems, because in the previous article [13] only preliminary MDs simulations were performed on the interaction with a single peptide and PD-L1 protein in solution state. In this way, the aggregation effect of the peptides in a monolayer and the role of the linker and of the spacer when bound to NSs in ruling the interaction with PD-L1 were both discarded.

In previous articles [11,12], we report that MDs simulations on NSs carrying engineered GE11 or cRGD demonstrated that the insertion of a charged spacer in the linker between the peptide and NP influences, for example, the exposure of peptides to water and thus to the antigen present in a tumor cell. Furthermore, the presence of the linker increases the flexibility and mobility of the peptide bound on the nanoparticles. However, in these works no explicit molecular interaction of NSs with the receptor was studied.

In the present article, the behaviors of NS systems that are properly functionalized with CLP002 or with its scrambled version SCLP002 in the absence and also in the presence of PD-L1 are understood by means of MDs simulations. In fact, MDs simulations of monolayers of peptides bound to the gold surface have been performed in the presence of the extracellular portion of the PD-L1 protein. These results give evidence of the different targeting activities observed experimentally [13] and suggest the key parameters that govern the interaction of the nanostructures with the target antigen.

In detail, MDs simulations were performed for a functionalized NP containing conjugated peptides (Table 1) and allowed for the investigation of both the influence of the primary sequence of the peptide (CLP002 or SCLP002) and of the linker (C- or P-) on the targeting activity of NSs. The decision to conduct experiments exclusively with functionalized peptides at the N-terminal, rather than the C-terminal, was based on the results of the MD simulation in solution which were reported elsewhere [13]. Briefly, these simulations revealed that amino acids in the C-terminal region exhibit a greater tendency to interact with the target (PD-L1) in a more extensive region. Consequently, to enhance exposure and facilitate potential interactions with the target, the N-terminal functionalized peptides were synthesized and studied.

The acronyms NS@P-CLP, NS@P-SCLP, and NS@C-CLP are used to indicate nanostructures functionalized with P-CLP, P-SCLP, and C-CLP, respectively (Table 1). The presence of the thiol group in the P-linker and of Cys in the C-linker allows the formation of the Au-S bond and thus the peptides, functionalized with a linker, to be covalently bound on the gold surface.

## 2. Results and Discussion

The results of the targeting activity on MDA-MB-231 cells, from human breast adenocarcinoma, which overexpress PD-L1, show that NSs functionalized with CLP002 (see Table 1), which are NS@C-CLP and NS@P-CLP, are more active than those carrying SCLP002 (NS@P-SCLP), as reported in Figure S1.

At the same time, interestingly, a different targeting activity is observed between NSs that carry CLP002, depending on the linker, with NS@C-CLP being much less active than NS@P-CLP. This means that the presence of CLP002 on NSs does not ensure the effectiveness of nanosystems, but the nature of the linker also plays a key role. In fact, to obtain a good targeting activity, the longer P-linker is more effective than the shorter C-linker present in the NS@C-CLP system.

Therefore, to obtain information on an atomistic scale of key features that ensure an effective targeting activity, MD simulation concerning the binding of PD-L1 to a designed peptide is not sufficient. Indeed, in our case, a recent article [13] has demonstrated through MDs calculations that CLP002 in solution, in its monomeric form, can recognize the binding region where the antigen PD-L1 interacts with the PD-1 protein, whereas this is not observed for SCLP002.

As explained earlier, the determination of the interaction that leads to CLP002/PD-L1 recognition in solution can only be considered as the first indication of what happens when many peptides are clustered on the gold surface. A more tuned approach involves the MDs simulation of the interaction of PD-L1 with NSs. To this end, MDs simulations of NS@C-CLP, NS@P-CLP, and NS@P-SCLP were performed using a gold surface, mimicking the surface of the NP, carrying a suitable number of peptides covalently bound to the gold via their linker.

### 2.1. The Role of the Primary Structure

One of the critical points in designing a theragnostic peptide assembly is specificity. To this end, to evaluate the specificity of CLP002 for PD-L1 we have already shown that a peptide (SCLP002) with the same amino acids but a scrambled sequence shows low targeting ability (Figure S1) in MDA-MB-231 cells. In this article, MDs simulations on these systems have been performed to unravel this result.

**Figure 1:**
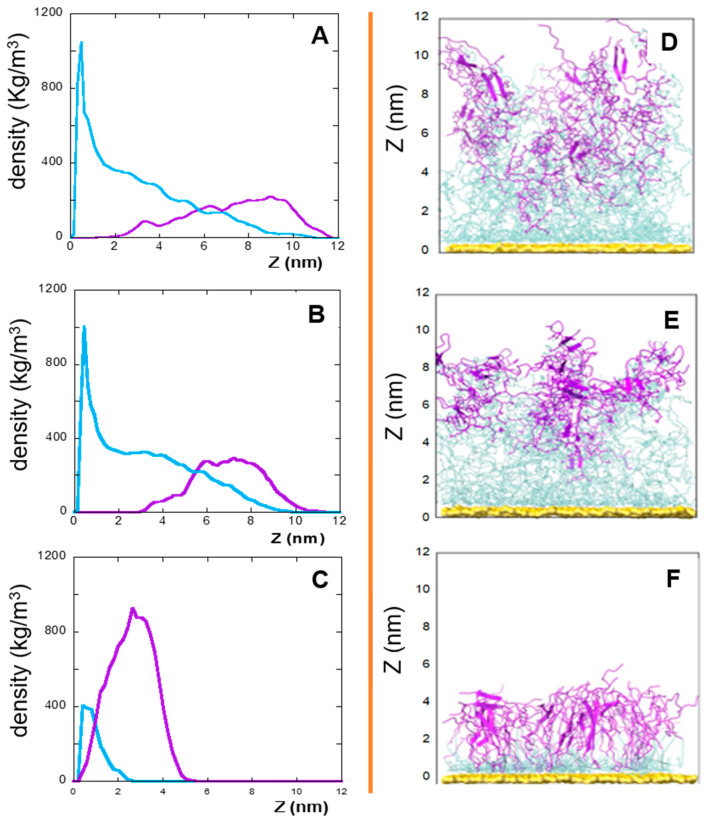
Left: densities along the Z-axis of linker (light blue) and peptide (violet) moieties for: P-CLP (**A**), P-SCLP (**B**), and C-CLP (**C**) on the NS surface in the last 20 ns of the simulations. Right: side view of NS@P-CLP (**D**), NS@P-SCLP (**E**), and NS@C-CLP (**F**) on the gold surface. The gold surface is yellow, the linker is light blue, and peptide moiety is violet.

In this context, the densities (versus the distance from the gold surface), of the linker and peptide moieties of the monolayer assemblies in NS@P-CLP and NS@P-SCLP are reported in Figure 1A,B. First, the higher density portions on the NS coating components are compatible with the thicknesses of the NS corona found in the TEM images [34] reported in Figure S2. In fact, they show that for NS@P-CLP and NS@P-SCLP a corona is formed around the coated nanoparticles, the thickness of which is 2.5 ± 0.4 nm and 2.1 ± 0.4 nm, respectively. This is an important result that indicates that the peptides each carrying a suitable linker formed a monolayer on the NS surface, which confirms the suitability of our MD model. In Figure 1D, one can see that the peptide moieties of P-CLP form, on the gold surface, a less compact structure than that of P-SCLP (Figure 1E). In fact, the peptide CLP002, as shown in Figure 1A, is distributed along the Z-axis (perpendicular to the gold surface) for 10 nm, indicating that some peptides are close to the surface, about 2 nm from it, and others protrude toward bulk water (at about 12 nm from gold).

This is not the case for the peptide component of NS@P-SCLP, which is mainly localized in a region which is approximately 5 nm long and which is from 5 to 10 nm from the gold surface (Figure 1B). This distance is only slightly higher than the peptide length (approximately 4 nm if in the elongated state), and thus the peptides assume a more compact structure.

**Figure 2:**
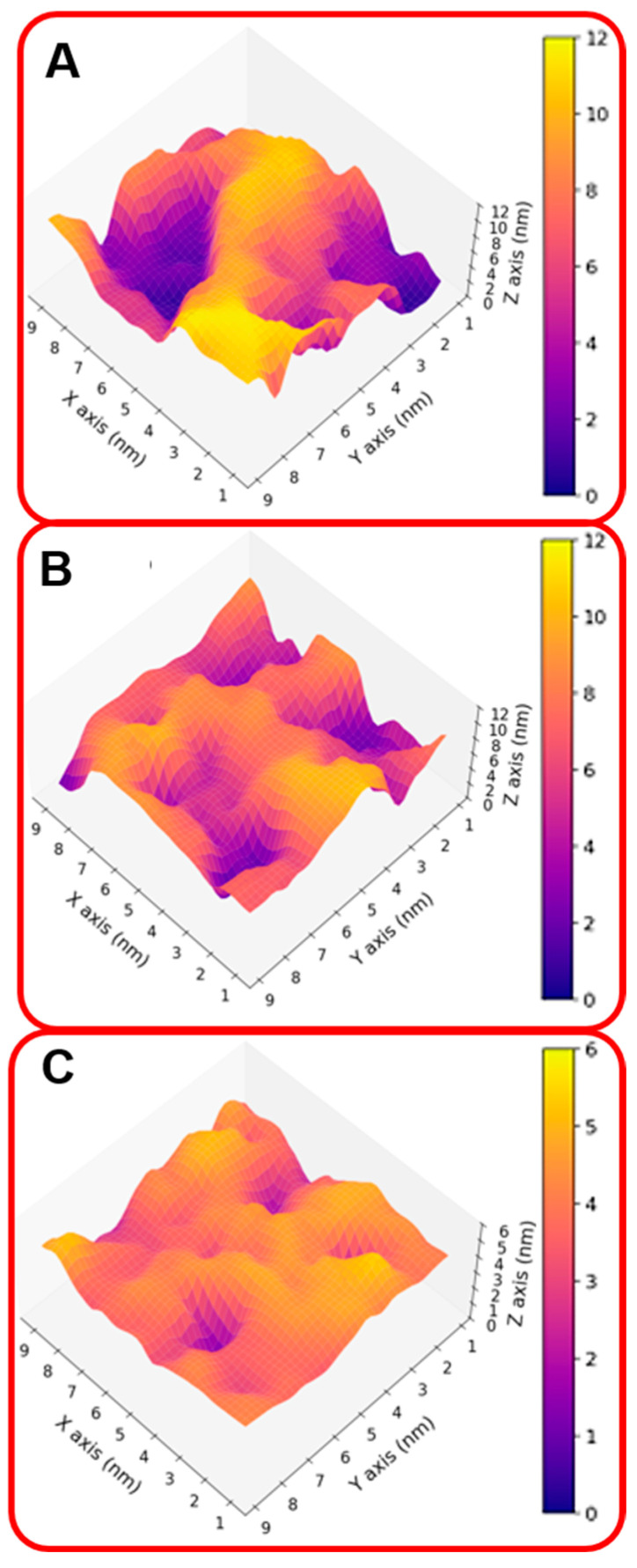
Three-dimensional representation of the monolayer surface of NS@P-CLP (**A**), NS@P-SCLP (**B**), and NS@C-CLP (**C**).

These results indicate that the organizations of the molecular components on the gold surface appear very different. This can be better seen in Figure 2A,B where the surfaces are shown in a three-dimensional representation. In particular, in the case of NS@P-CLP (Figure 2A), the peptide aggregates form a rough surface characterized by large and deep pockets in which the IgV-like domain of PD-L1 [35] (with a depth of 4 nm and a width of 3 nm; see Figure S3) can be easily accommodated.

In the case of NS@P-SCLP, the formation of a more compact peptide structure leads, as reported in Figure 2B, to a grid on the surface with relatively narrow meshes, making the complete insertion in the IgV-like domain less probable.

However, it is important to note that the performance of a system cannot be reduced to a purely geometric effect. In fact, as will be described later, for valuable affinity it is also necessary to have a correct orientation that allows for the specific interaction between the active region of the IgV-like domain of PD-L1 and the peptide residues present in the monolayer.

**Figure 3:**
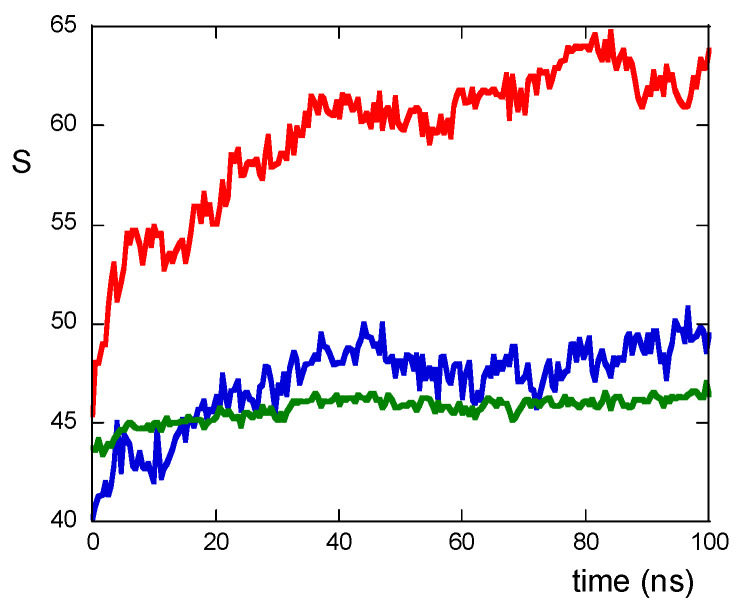
S function values for NS@P-CLP (red), NS@P-SCLP (blue), and NS@C-CLP (green) during MD simulation.

The peptide–peptide interactions can be described by counting the number of contacts among atoms of residues belonging to different peptides and are quantifiable via the S function (see Materials and Methods section). As shown in Figure 3, the values of the S function are lower for NS@P-SCLP than for NS@P-CLP; this indicates that the scrambled sequence shows a lower number of interactions among residues belonging to different peptides, leading to a grid formation of peptides in the monolayer. In the case of NS@P-CLP, instead, the peptides interact more with each other, forming relatively large clusters, thus leading to zones with high peptide density.

**Figure 4:**
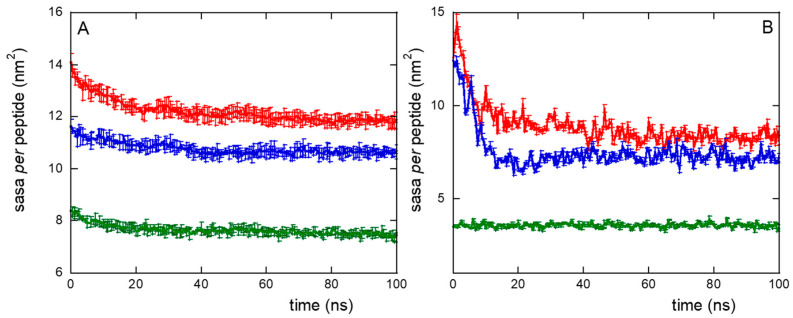
Surface accessible solvent area (SASA) per peptide using a probe radius of 0.15 nm (**A**) or 1.5 nm (**B**), for NS@P-CLP (red), NS@P-SCLP (blue), and NS@C-CLP (green), during MD simulation. Data are the averages, with relative error bars, of the two replicas performed.

The formation of peptide clusters in NS@P-CLP compared to that in NS@P-SCLP was also confirmed by surface accessible solvent area (SASA) measurements. The SASA values for CLP002, using a probe of radius of 0.15 nm (mimicking water accessibility) and a probe of 1.5 nm (mimicking PD-L1 accessibility, as it is close to its active domain dimension, the IgV-like one, Figure S3), are approximately 15% higher than those obtained with SCLP002 (Figure 4A,B). It should be considered that this difference refers to the single peptide and gives rise to a total SASA of 63 nm^2^ more for P-CLP than for P-SCLP in the case of the monolayer portion simulated by us (approximately 10 × 10 nm^2^). If, for example, we consider the surface of a small nanoparticle with a radius of 10 nm, the difference between the two systems becomes about 780 nm^2^, that is, more than ten times the surface of the IgV-like domain of PD-L1 (75 nm^2^). Even if there is no direct correlation between SASA and binding with PD-L1, a higher SASA suggests greater ease of penetration into the grooves of the monolayer, increasing the probability that the active region will contact the appropriate residues of the monolayer peptides. Therefore, the SASA results, together with the presence of depressions that can allocate the active domain of PD-L1 (Figure 2), indicate a higher accessibility of the peptide moiety to water and PD-L1 for the NS@P-CLP system.

In summary, the linker for the P-CLP and P-SCLP systems is particularly long and mobile. In the initial phases of the simulations, the long PEG chains tend to curl and become closer to the gold surface. The peptide fractions instead interact with each other through contact between hydrophobic groups and between polar or charged sidechains and H-bonds. Therefore, the equilibrated systems are made up of peptide aggregates that ‘float’ on the PEG phase closest to the surface (see Figure 1A–C). The structure of the aggregates also influences the PEG phase and contributes to the formation of more or less deep depressions.

**Figure 5:**
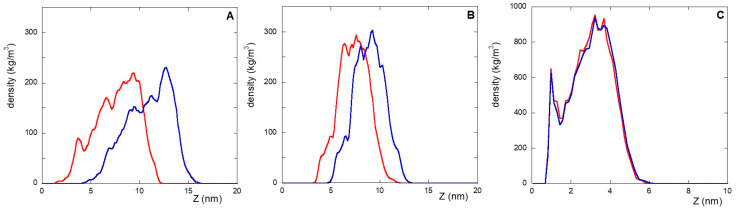
Displacement, along the Z-axis, of the peptide moiety of NS@P-CLP (**A**), NS@P-SCLP (**B**), and NS@C-CLP (**C**) before (red) and after (blue) application of a constant stretching force for 10 ns.

The different organization of the monolayer components for NS@P-CLP and NS@P-SCLP described above also explains the different deformation properties of the monolayers demonstrated by a simulated tensile test carried out using MD simulations lasting 10 ns. Higher deformation capability suggests a higher mobility of the monolayer to manage the moieties (linker and peptide) to better accommodate the IgV-like domain of PD-L1. A constant stretching force (acceleration equal to 0.01 nm/ps^2^) on the C^α^ atoms of the peptides causes a higher deformation for NS@P-CLP (Figure 5A) than for NS@P-SCLP (Figure 5B). The average density of the monolayer, indeed, is shifted along the Z-axis, perpendicular to the gold surface of 1.31 ± 0.06 nm and 0.71 ± 0.03 nm for NS@P-CLP and NS@P-SCLP, respectively. These data indicate that the mobility under the deformation action (as in the case with the insertion of PD-L1 into the monolayer) is different for the two coatings, which gives further suggestions that in the case of the NS@P-CLP monolayer the peptides are more able to form a binding site capable of hosting the IgV-like domain of PD-L1.

**Figure 6:**
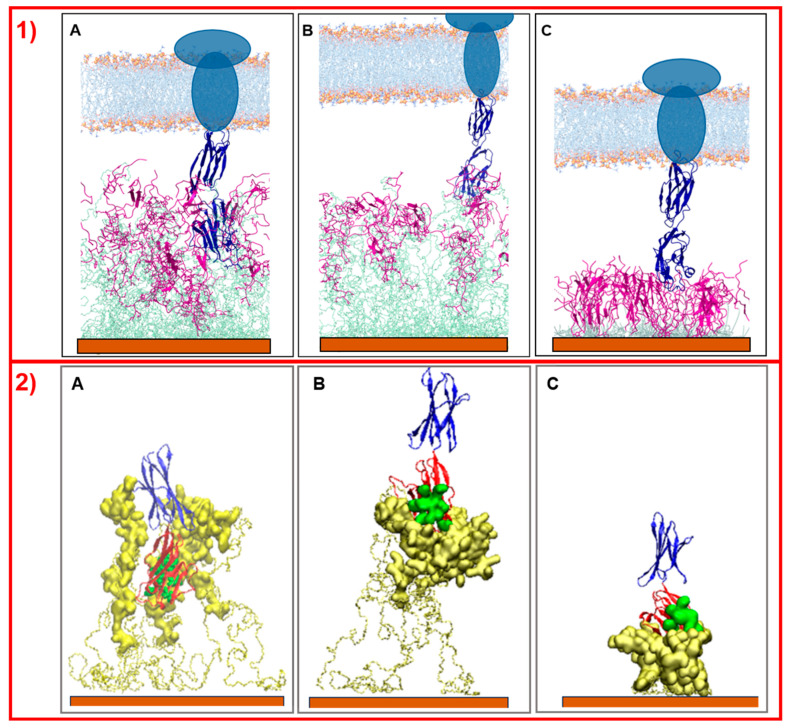
Side view picture of the last frames of the simulations of PD-L1 with NS@P-CLP (**A**), NS@P-SCLP (**B**), and NS@C-CLP (**C**). In panel (**1**) the two linked extracellular domains of PD-L1 are blue, the peptide moieties are violet, the linker moieties are light green, and the gold surface is orange. The membrane (depicted in light blue and orange) and the intermembrane/intracellular portions of PD-L1 (dark blue ellipses) are schematically shown in the upper part of the images. In panel (**2**), only the engineered peptide components that participate in the binding and extracellular domains of PD-L1 are depicted. The gold surface is orange, the linkers are represented by yellow bonds, the peptides by yellow surface, and the IgV-like domain of PD-L1 is in red, while its active residues are green spheres and the second domain of PD-L1 is blue.

Simulations of the interaction of NS@P-CLP and NS@C-CLP with PD-L1 elucidate why the first system is more active than the last. As reported by Zak et al. [14], the PD-L1 region of contact with PD-1 is located in one of the two extracellular domains (the IgV- like domain) of the antigen. As shown in Figure 6, only in the case of NS@P-CLP systems, these PD-L1 domains are within the monolayer assembly and they interact strongly with the peptide moieties (Figure 6A), while for NS@P-SCLP the interaction appears to be weak (Figure 6B). This is also evidenced by the calculation of the binding energies of PD-L1 and monolayers performed according to Thomas and Dill [36,37], which is reported in Figure S4. In fact, the number of close contacts between PD-L1 and the peptide monolayer is found to be 1473 ± 147 and 1079 ± 138 for NS@P-CLP and NS@P-SCLP, respectively, which confirms that the strongest interaction is observed for NS@P-CLP.

Furthermore, the binding between PD-L1 and the peptides was analyzed in detail in the region of contact between PD-L1 and PD-1. This contact region presents several residues in the PD-L1 IgV-like domain, called active spots (ASs), and comprises hydrophilic and hydrophobic PD-L1 residues, namely: Phe19, Asp26, Ile54, Tyr56, Gln 66, Arg113, Met115, Ala121, Asp122, Tyr123, Lys 124, and Arg125 [13,23]. The number of contacts among the peptides in the monolayers and the active spots in the last 20 ns of simulation is found to be 517 ± 53 and 89 ± 12 for NS@P-CLP and NS@P-SCLP, respectively.

**Figure 7:**
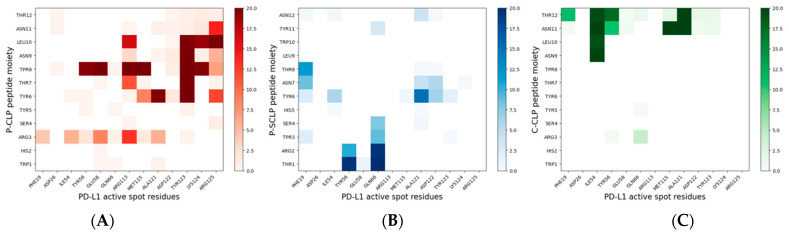
Bidimensional histograms of the persistence time of contacts between peptide residues and active spot residues of PD-L1, for NS@P-CLP, (**A**), NS@P-SCLP (**B**), and NS@C-CLP (**C**). The sidebar of each histogram provides a quantitative evaluation of the persistence time of each contact.

In more detail, in the contact maps shown in Figure 7 the persistence times of contacts between a peptide residue and the residues of the active region of the IgV domain of PD-L1 are reported as a two-dimensional histogram when the systems are in equilibrium (in the last 20 ns of simulations). As shown in the figure, in the NS@P-CLP system all active spots interact with at least one peptide residue and many contacts persist for the entire duration analyzed. In the case of the NS@P-SCLP system, the numbers of contacts are significantly smaller and mainly involve the N-terminal residues for the former and the C-terminal residues for the latter. To better visualize this interaction, in Figure 6 panel 2 and in Figure S5, the details of the last frame of the MDs simulations regarding PD-L1 and the functionalized NP are reported. These figures clearly show that the active spots of PD-L1 interact with the peptides only for NS@P-CLP, confirming that the active region of PD-L1 plays a fundamental role in the binding to NS, in agreement with the experimental activities of the different systems studied, as reported in Figure S1.

### 2.2. The Role of the Linker

In this work, an alternative is presented to the well-established coupling of the peptide to a thiolate PEG chain via a cationic pentapeptide spacer (Lys_3_Gly_2_), which increases the accessibility of the targeting peptide on the nanoparticle surface and prevents proteolytic degradation [10,12,19]. In the NS@C-CLP002 system, in fact, a short oligoethylene linker was introduced between the CLP002 sequence and the terminal cysteine residue to increase the flexibility and mobility of the peptide bound to the nanoparticles (see Table 1). In this way, the role of the linker in PD-L1 binding is evaluated. This can be obtained by comparing the characteristics of NS@P-CLP and NS@C-CLP, which carry the same active peptide (CLP002) but are linked to the NP with a different linker (P- or C-). First, the higher density portion of the engineered peptide components as a function of the distance from the gold surface is also in this case, as shown in Figure 1C, very close to the corona depth of 3.4 ± 0.3 nm, as found by TEM experiments (Figure S2C).

This confirms that the engineered peptides form a monolayer on the NP surface. Frames at the end of the MDs simulations in NS@P-CLP and NS@C-CLP are reported in Figure 1D,F. It is interesting to note that the arrangement of the molecular components on the gold surface appears very different. In particular, as shown in Figure 1A and Figure 2A, NS@P-CLP forms an irregular surface characterized by deep pockets (see Section 2.1). In the case of NS@C-CLP, the peptides form a uniform compact layer characterized by a constant peptide density along the Z-axis which is only slightly higher (approximately 5 nm) than the peptide length (approximately 4 nm if in the elongated state) with almost no void space (Figure 1C and Figure 2C). Obviously, the presence of a compact layer makes the interaction with the IgV-like domain of PD-L1 unlikely as the surface cannot contain the entire domain and therefore its interaction can only occur with limited regions of it. These different features between NS@P-CLP and NS@C-CLP are attributable to the role of the PEG moiety in the P-linker, which allows for higher mobility of the peptides in the monolayer and greater peptide aggregation capability. As shown in Figure 3, in fact, the number of contacts between residues belonging to different peptides, during the MD simulation, is higher for NS@P-CLP than for NS@C-CLP, indicating that the CLP002 units in NS@P-CLP form more compact clusters in NS@P-CLP than in NS@C-CLP. The formation of larger peptide aggregates in NS@P-CLP compared to NS@C-CLP was also assessed by SASA measurements. In fact, the SASA values for NS@C-CLP obtained using a probe with a radius of 0.15 nm (Figure 4A) are almost half of those for NS@P-CLP during all the simulation time. Similar results have been obtained using a larger SASA probe (radius: 1.5 nm; Figure 4B). These results reveal, for NS@C-CLP, the formation of a compact structure throughout the monolayer, suggesting low accessibility for PD-L1.

Furthermore, it should be noted that while in the case of NS@C-CLP the SASA values are almost constant during the simulation time, indicating the absence of a substantial rearrangement of the peptide monolayer, this is not the case for NS@P-CLP due to the greater mobility of the P- linker which leads to equilibrium after approximatively 10–20 ns.

This characteristic further underlines the fundamental role of PEG in the overall monolayer features. The compactness and high rigidity of the engineered peptide monolayer of NS@C-CLP have also been assessed by an MD simulated tensile test (Figure 5); in this case, as shown in Figure 5A,C, unlike NS@P-CLP almost no deformation is observed under a constant stretching force (the average weighted density is shifted along the Z-axis, perpendicular to the surface, of 0.15 ± 0.01 nm). This result suggests a very low deformability of the monolayer, which is unable to ‘accommodate’ and interact with PD-L1.

Simulations of this nanostructure with PD-L1 show very low interaction between the protein and the monolayer (Figure 6C), while, as explained earlier, PD-L1 is well-inserted into the assembly of the NS@P-CLP peptide. In fact, the number of close contacts per peptide between PD-L1 and the peptides in the monolayer for NS@C-CLP is almost half (882 ± 90) compared to the number for NS@P-CLP. Furthermore, the binding energy (Figure S4) is also negligible and, more importantly, those between AS and CLP002 in NS@C-CLP are more than three times lower (155 ± 24) than in NS@P-CLP (see also Figure 7C and Figure S5) and involve mainly the C-terminal residues of the peptides. Furthermore, these data confirm the higher affinity between PD-L1 and NS functionalized with CLP002 peptides linked to NS with a P-chain. Therefore, in general, these results give a good interpretation of the experimental data on the activities (see Figure S1) of the NSs.

## 3. Materials and Methods

### 3.1. Functionalized Nanostructures Synthesis and Characterization by Transmission Electron Microscopy Measurements

Peptides, relative conjugates (Table 1), and gold NPs were synthesized as reported elsewhere [13]. Furthermore, the functionalization and characterization of NSs with conjugated peptides were performed following the protocol already reported [4,6].

The morphology and microstructure of the samples were characterized by transmission electron microscopy (TEM) using a TEM JEOL F200 (JEOL Ltd., Tokyo, Japan). The images were recorded using an accelerating voltage of 200 kV. Carbon-supported copper grids, 400 mesh size, were used for preparation of the sample.

### 3.2. MDs Simulations

MDs simulations were carried out using Gromacs software package version 2020.6. The Gromos53a6 force field [38] was used with the addition of the parameters relating to the PEG portion [39] and the Au atom, [40] as already reported [11].

For all simulations, an explicit simple point charge (SPC) water molecule was used, and chloride ions were added to ensure electroneutrality.

For all the systems investigated, two replicas of 100 ns each were performed. A constant pressure and temperature ensemble (NPT) was used for all simulations performed, with periodic boundary conditions. For each system, the energy equilibrated for 100 ps MD at 300 K using a timestep of 0.5 fs was first minimized. Production runs were performed, using a time step of 2 fs. The particle mesh Ewald (PME) algorithm [41,42] was used for the electrostatic interactions (cut-off = 1.4 nm). A cut-off was also used for the van der Waals interactions (1.4 nm). In all simulations, the velocity rescale scheme [43] was used to keep temperature constant (coupling constant σ_T_ = 0.6 ps) and the Berendsen algorithm [44] under isotropic conditions for pressure coupling (t_P_ = 1 ps).

The initial gold surface monolayer was obtained by placing the Au atoms in a hexagonal close-packed arrangement. Subsequently, the energy of this monolayer was minimized by applying a harmonic constraint with a force constant equal to 10,000 kJ mol^−1^ nm^−2^ on all the atom positions. This minimized gold monolayer was used for all simulations involving systems absorbed on the gold surface.

The systems were obtained by inserting the maximum number of molecules compatible with the simulation box (Table 2). For all systems, the starting molecular structure contains the most stable conformation of the peptide, obtained from aqueous simulations whose results have already been reported [13], and the all-trans conformation was considered for the remaining portions. To obtain the starting configuration of each monolayer, the molecules were inserted with the long axis parallel to the Z-direction (i.e., perpendicular to the surface) with the S atoms of the thiolate PEG (P-CLP and P-SCLP) or Cys (C-CLP) close (0.294 nm) to the Au layer.

To study the interactions between the target and each peptide monolayer, MDs simulations of 100 ns (two replicas) were performed on the system containing the final monolayer structure and the PD-L1 protein [pdb code: 3BIK]. The convergence of the simulation has been assessed by monitoring over time the average of the minimum distances of the side chains of the peptides that in the last 20 ns of the simulation have at least one heavy atom at a distance less than 0.7 nm from PD-L1 (Figure S5). Furthermore, we have followed, for a period of time (at time intervals of 20 ns), the value of the distance from the surface at which the peptide monolayer reaches a density of 200 kg/m^3^. The convergence of this general parameter also confirms the achievement of the equilibrium condition (Figure S6).

The S function (Equation (1)) [45] was used to obtain the number of contacts among the peptide residues, during the simulations. The function is as follows:

(1) $$S=\sum_{i}\sum_{j}\frac{{1-\left(\frac{r_{ij}}{r_{0}}\right)}^{6}}{{1-\left(\frac{r_{ij}}{r_{0}}\right)}^{14}}$$

where r_ij_ is the distance between two atoms i and j belonging to residues of different peptides, r_0_ is 0.6 nm, and 6 and 14 are exponents that allow the smoothness of the function. The S values per peptide, shown in Figure 3, have been normalized for n(n-1)/2, where n is the total number of peptides.

The surface accessible solvent area (SASA), conformational clusters, and density were obtained using the corresponding Gromacs tools [46]. In detail, the density was obtained by dividing the simulation box into slices, with a thickness equal to 0.15 nm, as a function of the distance Z from the gold surface. To evaluate the close contact between residues, we have considered all atoms of different molecules at a distance less than 0.5 nm in the last 20 ns of MD simulation.

All of the parameters and simulations data analyzed have been obtained in the last 20 ns of each simulation time, when the systems are equilibrated, and finally only the average values of the replicas were reported.

The visual molecular dynamics (VMD, version 1.9.3) program [47] was used for structure visualization.

## 4. Conclusions

MDs simulations allowed us to obtain details of the interactions of gold NSs functionalized with the CLP002 peptide, which was engineered with the P-linker with the immune checkpoint PD-L1. It has been demonstrated that targeting is due to a complex interplay between the presence of peptides that carry a primary sequence that can interact with the hot spots of PD-L1 and an appropriate chain to link the peptide to NSs. In other words, functionalization with the CLP002 peptide is a necessary but not sufficient condition for targeting. In fact, NS@C-CLP shows much lower targeting activity than NS@P-CLP. As demonstrated by MDs simulations, a longer P-linker ensures proper mobility and fluidity of the organic coating and allows the peptide moieties to rearrange and aggregate, forming deep pockets in which PD-L1 can insert and interact. This is not possible in the presence of the C-linker because the peptides form an almost compact and un-deformable layer on the NSs, thus preventing a valuable interaction with PD-L1. Furthermore, the primary sequence of the peptide is also fundamental for targeting. This was experimentally shown with NS@P-SCLP [13] and was justified by MDs results.

## Figures and Tables

**Table 1:** Peptides, linkers, conjugated peptides (linker and peptides), and relative acronyms used in the functionalization of NSs.

Peptide	Sequence ^1^
CLP002	Trp-His-Arg-Ser-Tyr-Tyr-Thr-Trp-Asn-Leu-Asn-Thr
SCLP002	Thr-Arg-Trp-Ser-His-Tyr-Asn-Thr-Leu-Trp-Tyr-Asn
**Linker**	
C-	Ac-Cys-(O2oc)_2_-
P-	HS-PEG-Lys_3_-Gly_2_-
**Linker and peptide**	
C-CLP	Ac-Cys-(O2oc)_2_-CLP002-NH_2_
P-CLP	HS-PEG-Lys_3_-Gly_2_-CLP002-NH_2_
P-SCLP	HS-PEG-Lys_3_-Gly_2_-SCLP002-NH_2_

^1^ Standard abbreviation for natural amino acids. Ac: acetyl group; O2oc: -NH(CH_2_CH_2_O)_2_CH_2_-CO-; PEG: polyethylene glycol of MW = 3000 Da (PEG_3000_).

**Table 2:** Number of peptides, size of the boxes, number of added H_2_O molecules, and ions (Cl^-^) in the MD simulation.

System	Box (nm^3^)	Peptides	Water	Ions (Cl^−^)
NS@P-CLP	10.32 × 9.99 × 27.55	45	79,050	180
NS@P-SCLP	10.30 × 9.97 × 27.50	45	78,349	180
NS@C-CLP	10.31 × 9.98 × 18.17	90	50,807	90

## Data Availability

The data that support the findings of this study are available on the online repository Zenodo (https://doi.org/10.5281/zenodo.15333493).

## Supplementary Materials

The following supporting information can be downloaded at: https://www.mdpi.com/article/10.3390/molecules30092045/s1. Figure S1: Targeting Activity of the SERS-NS systems against MDA-MB-231 cells. Figure S2: TEM images of SERS-NS systems. Figure S3: Scheme of PD-L1 highlighting the diameter of the portion interacting with PD1. Figure S4: PD-L1/NS Binding energy as a function of MD simulation time. Figure S5: Side view of the last frame of MD simulation on the interaction of PD-L1 with SERS-NS systems. Figure S6: Average minimum distances, over time, between the side chains of peptides interacting with PD-L1 and the protein for NS systems. Figure S7: Distance from the surface at which in the presence of PD-L1, the peptide monolayer of NS system reaches a density of 200 kg/m^3^.
