# Supplementary material for: Unraveling the Specific Recognition Between PD-L1 and Engineered CLP002 Functionalized Gold Nanostructures: MD Simulation Studies
Source: Molecules. 2025 May 4;30(9):2045. doi: 10.3390/molecules30092045 (PMC12073790; doi:10.3390/molecules30092045)
Supplement: Supplementary file 1 [file molecules-30-02045-s001.zip › molecules-3504169-supplementary - Copy.pdf]

molecules

MDPI

SUPPLEMENTARY MATERIALS

Unraveling the specific recognition between the PD-L1 11and functionalized CLP002-based gold nanoparticles: MD simulation studies.

Micaela Giannetti[1], Marina Gobbo[2], Lucio Litti[2], Isabella Caligiuri[3], Flavio Rizzolio[3,4], Moreno Meneghetti[2], Claudia Mazzuca[1*], and Antonio Palleschi[1*]

[1] Department of Chemical Science and Technologies, University of Rome "Tor Vergata", Via della Ricerca Scientifica, 00133 Rome, Italy
[2] Department of Chemical Sciences, University of Padova, Via F. Marzolo 1, 35131 Padova, Italy
[3] Pathology Unit, Centro di Riferimento Oncologico di Aviano (CRO) IRCCS, Via F. Gallini 2, 33081 Aviano, PN, Italy
[4] Pathology Unit, Department of Molecular Sciences and Nanosystems, Ca' Foscari University of Venice, Via Torino 155, 30172 Venice, Italy
* Correspondence: claudia.mazzuca@uniroma2.it (C.M.); antonio.palleschi@uniroma2.it (A.P.); Tel.: +39 06 72594467 (C.M.); +39 06 72594466 (A.P.)

Results and Discussion

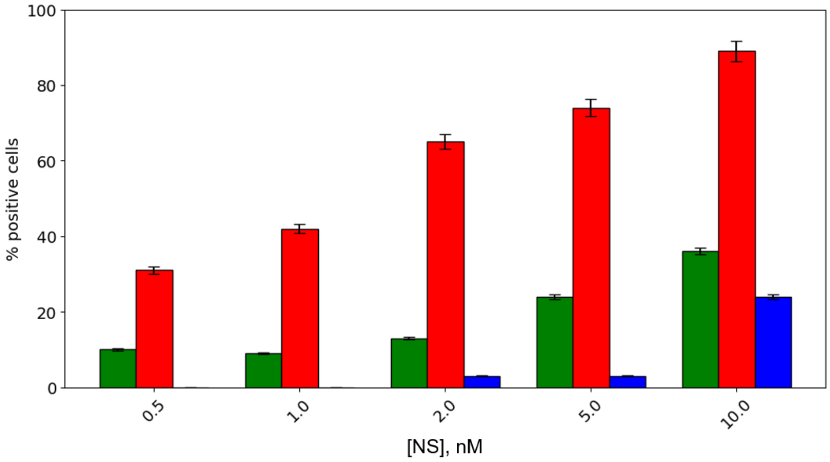

Figure S1. Targeting activity of the SERS-NS systems against MDA-MB-231 cells: NS@P-CLP (red), NS@P-SCLP (blue), NS@C-CLP (green) *(adapted from [7]).*

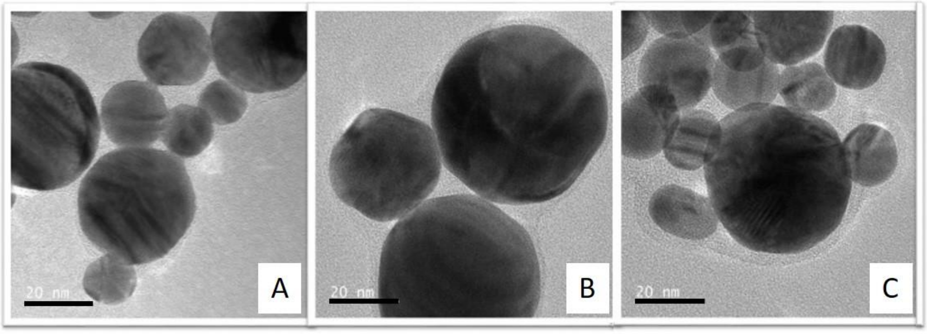

Figure S2. TEM images of NS@P-CLP (A), NS@P-SCLP (B), NS@C-CLP (C). The scale bar is 20 nm.

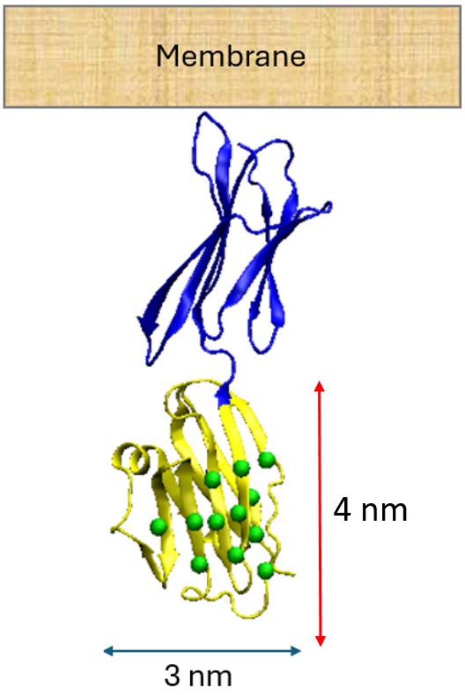

Figure S3. Scheme of PD-L1 highlighting the dimensions estimated by X-ray structure reported in pdb code: 3BIK, of the portion (yellow,) interacting with PD1 (that is the IgV-like domain): amino acids responsible for the interaction (active spots, see main text) are in green.

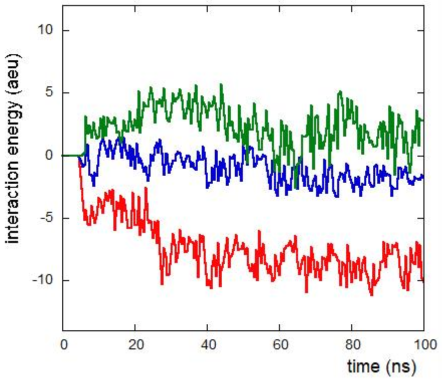

Figure S4. Binding energy as a function of MD simulation time with PD-L1 for NS@P-CLP (red), NS@P-SCLP (blue) and NS@C-CLP (green).

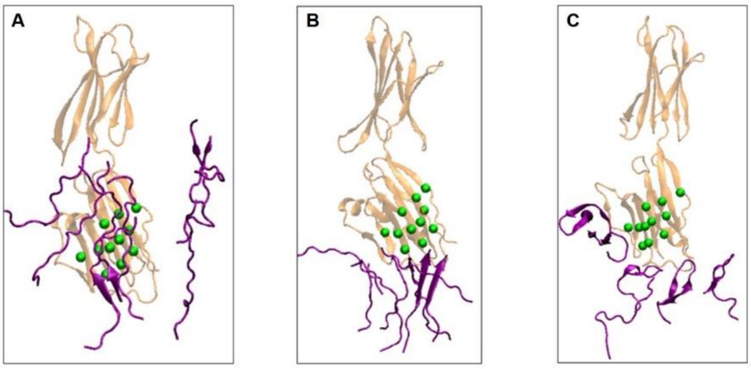

Figure S5. Side view of the last frame of MD simulation on the interaction of PD-L1 with NS@P-CLP (A), NS@P-SCLP (B), NS@C-CLP (C). PD-L1 is beige, peptides are violet; active spot residues of PD-L1 are green.

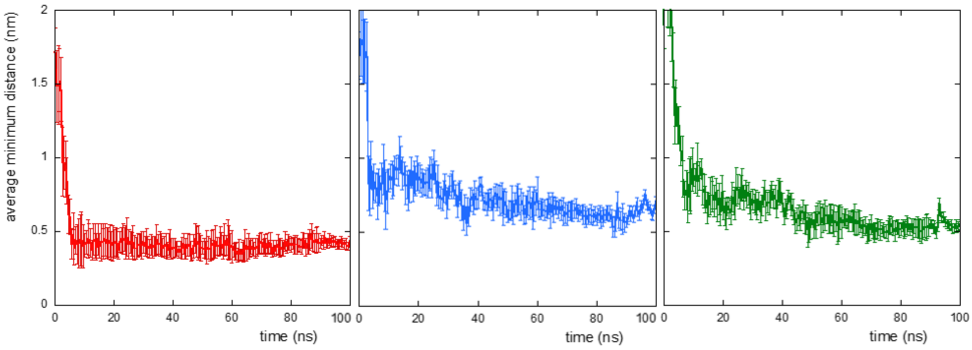

Figure S6. Average minimum distances, over time, between the side chains of peptides interacting with PD-L1 and the protein for NS@P-CLP (red), NS@P-SCLP (blue) and NS@C-CLP (green).

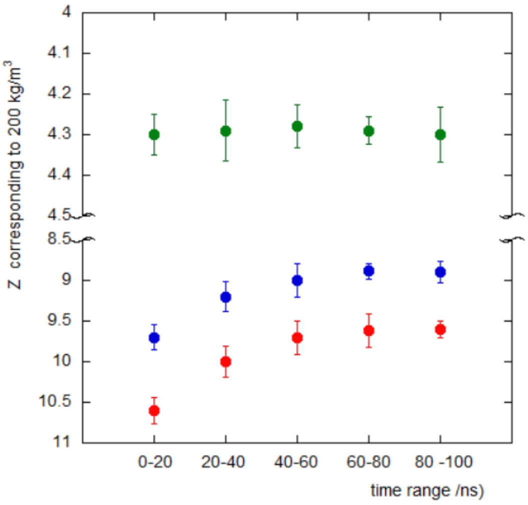

Figure S7. Distance from the surface (that is along the Z axis) at which in the presence of PD-L1, the peptide monolayer of NS@P-CLP (red), NS@P-SCLP (blue) and (NS@C-CLP (green) reaches a density of 200 kg/m^3.
